# A single-institution experience with ^177^Lu RPT workflow improvements and qualifying the SPECT/CT imaging for dosimetry

**DOI:** 10.3389/fonc.2024.1331266

**Published:** 2024-02-26

**Authors:** Siju C. George, Ranjini Tolakanahalli, Santiago Aguirre, Taehyung Peter Kim, E. James Jebaseelan Samuel, Vivek Mishra

**Affiliations:** ^1^ Department of Radiation Oncology, Miami Cancer Institute, Baptist Health, Miami, FL, United States; ^2^ Department of Physics, School of Advanced Sciences, Vellore Institute of Technology, Vellore, India

**Keywords:** ^177^Lu treatments, patient-specific dosimetry, clinical implementation, dose calibration, registration error, treatment day checklist, clinical workflow, hybrid imaging system QA

## Abstract

**Background and purpose:**

Implementing any radiopharmaceutical therapy (RPT) program requires a comprehensive review of system readiness, appropriate workflows, and training to ensure safe and efficient treatment delivery. A quantitative assessment of the dose delivered to targets and organs at risk (OAR) using RPT is possible by correlating the absorbed doses with the delivered radioactivity. Integrating dosimetry into an established RPT program demands a thorough analysis of the necessary components and system fine-tuning. This study aims to report an optimized workflow for molecular radiation therapy using ^177^Lu with a primary focus on integrating patient-specific dosimetry into an established radiopharmaceutical program in a radiation oncology setting.

**Materials and methods:**

We comprehensively reviewed using the Plan–Do–Check–Act (PDCA) cycle, including efficacy and accuracy of delivery and all aspects of radiation safety of the RPT program. The GE Discovery SPECT/CT 670DR™ system was calibrated per MIM protocol for dose calculation on MIM SurePlan™ MRT software. Jaszcak Phantom with 15–20 mCi of ^177^Lu DOTATATE with 2.5 µM EDTA solution was used, with the main energy window defined as 208 keV ±10% (187.6 to 229.2 keV); the upper scatter energy window was set to 240 keV ±5% (228 to 252 keV), while the lower scatter energy window was 177.8 keV ±5% (168.9 to 186.7 keV). Volumetric quality control tests and adjustments were performed to ensure the correct alignment of the table, NM, and CT gantry on SPECT/CT. A comprehensive end-to-end (E2E) test was performed to ensure workflow, functionality, and quantitative dose accuracy.

**Results:**

Workflow improvements and checklists are presented after systematically analyzing over 400 administrations of ^177^Lu-based RPT. Injected activity to each sphere in the NEMA Phantom scan was quantified, and the MIM Sureplan MRT reconstruction images calculated activities within ±12% of the injected activity. Image alignment tests on the SPECT/CT showed a discrepancy of more than the maximum tolerance of 2.2 mm on any individual axis. As a result of servicing the machine and updating the VQC and COR corrections, the hybrid imaging system was adjusted to achieve an accuracy of <1 mm in all directions.

**Conclusion:**

Workflows and checklists, after analysis of system readiness and adequate training for staff and patients, are presented. Hardware and software components for patient-specific dosimetry are presented with a focus on hybrid image registration and correcting any errors that affect dosimetric quantification calculation. Moreover, this manuscript briefly overviews the necessary quality assurance requirements for converting diagnostic images into dosimetry measurement tools and integrating dosimetry for RPT based on ^177^Lu.

## 1 Introduction

^177^Lu radio pharmaceutical therapy (RPT) focuses on cancer cell destruction by targeting associated surface proteins and combining diagnostic and therapeutic capabilities. Notable applications are seen in Lutathera® and Pluvicto®, both administered as per FDA protocols through intravenous injections, with their dosages adapted in certain extenuating circumstances based on patient needs. The NETTER and VISION trials facilitated approval for these therapies, respectively, addressing neuroendocrine tumors (NET) and metastatic castrate-resistant prostate cancer (mCRPC) (1, 2). ^177^Lu RPT offers diagnostic and therapeutic features demonstrating versatility and increased safety. Medium-energy gamma photons aid precise imaging, while beta particles annihilate tumor tissues with minimal harm to the vital neighboring organs.

Clinical implementation of dosimetry in ^177^Lu-based RPT faces limitations due to a lack of tumor and normal tissue dose–response data. Standardized and accurate post-administration dosimetry can begin to fill in this data gap. Several challenges may be associated with incorporating dosimetry into an established RPT program. Non-uniform treatment protocols and differing patient-specific dosimetry practices in departments like radiation oncology and nuclear medicine add complexity. It is well known that RPTs target specific cells with radiation. Still, uncertainties in dose calibration, patient-specific biodistribution, and imaging data quality, as well as uncertainties in dosimetry models, remain.

Establishing standardized protocols and quality assurance procedures can minimize these uncertainties, affecting absorbed dose estimation (3, 4). Our study focuses on three vital technical aspects: standardizing dose measurement and delivery methods using appropriate checklists and workflows, qualifying the SPECT/CT images for dose conversion, and implementing patient-specific dosimetry software for RPT based on ^177^Lu with necessary validation tests. Standardization, harmonization, and automated techniques are crucial for enabling routine clinical implementation (5). Despite challenges stemming from program differences, achieving patient-specific dosimetry relies on collaborative efforts across nuclear medicine, diagnostic imaging, radiation physicists, and radiation oncologists, depending on program implementation at a specific institution.

^177^Lu post-treatment dosimetry utilizes various patient-specific dosimetry calculation programs, each employing distinct scientific principles and commercial implementation that use images collected using hybrid imaging systems. Dosimetry is the calculation of absorbed doses in various tissues and organs. Monte Carlo (MC) simulations may be precise enough for calculating s-factors for radiations emitted by radionuclides used in RPT. Instead of MC calculations, Voxel-s-value (VSV) or Local Deposition Methods (LDM) is expected to reduce calculation time and resource demand (6). Absorbed doses are then calculated from cumulated activities using well-established software packages. Many studies describe extensive details of dose quantification and uncertainties related to cumulative activities and energy deposition in ^177^Lu-based RPT (5, 7–10). Rather than delving deeper into patient-specific dosimetry calculations and uncertainties, this manuscript aims to suggest improvements to clinical workflow and image qualification, which is only the first step toward dose quantification with hybrid imaging followed by RPT administration. Integrating the appropriate imaging system into dosimetry software is essential for optimizing radiopharmaceutical therapy dosimetry (11, 12). The importance of accurate nuclear medicine quantification for therapy planning and the role of imaging in dosimetry are discussed in detail by Frey et al. (13). In addition, modern radiation therapy might require combination therapies or additional treatments for subsequent diseases, especially with external beam radiation therapy (EBRT), where post-treatment organ dose exposure data in the medical record are critical for clinical decision-making, (14).

This article describes our experience integrating a post-treatment dosimetry program for ^177^Lu RPT within a single institution, focusing on using GE SPECT/CT technology for image acquisition and MIM Sureplan MRT software for dose calculation. The paper is laid out as follows: In the first part, we review the workflows and checklists for setting up Pluvicto and Lutathera RPT programs to ensure program quality, smooth delivery, completeness, and optimization over 5 years of experience with Lu treatments in radiation oncology. After that, we will discuss the quality assurance and commissioning work related to imaging. We will focus on the hybrid image registration process, a critical initial step for integrating patient-specific dosimetry into an existing RPT program. In addition, we will provide a brief overview of the software integration and commissioning of a commercial dosimetry system, which is necessary for establishing a robust RPT program.

## 2 Materials and methods

### 2.1 A comprehensive review of the existing ^177^Lu-based RPT program

The Lutathera® RPT is administered over four cycles to NET patients, with an 8-week interval that may be extended to 16 weeks if toxicity issues arise. Before treatment, it's necessary to administer anti-emetic drugs for nausea and an amino acid solution rich in L-lysine and L-arginine to protect the kidneys. Lutathera® is intravenously delivered using vendor-specified infusion rates using an infusion pump. Patients get a 30-mg intramuscular long-acting release (LAR) octreotide injection post-infusion (15, 16). Pluvicto® treatment for mCRPC involves up to six bi-weekly doses, managing doses and adverse reactions as per medical protocols, and a mandatory 10-mL saline flush pre-administration, adopting institutional guidelines for extravasation cases. Using the Plan–Do–Check–Act (PDCA) tool, we reviewed workflows and treatment day checklists during and after the implementation of Pluvicto following 3 years of treatments with Lutathera. The PDCA process has four stages: Plan, Do, Check, and Act. Opportunities are identified, and an action plan is developed in the Plan stage. Changes are implemented on a small scale in Do, followed by assessing their impact in Check. Finally, based on the results, changes are implemented more broadly in the Act stage (17). Workflow, checklists, and critical highlights of additional safety measures implemented for the program are presented.

#### 2.1.1 Dose calibrator assessment

The nuclear medicine department’s QA program for the re-entrant chamber was evaluated. The department performs daily constancy checks to ensure the accuracy of activity measurements of the re-entrant chamber. Linearity check was performed quarterly (more frequently if indicated by the constancy check) by measuring the dose calibrator’s response to a series of sources with different activities and plotting them (18, 19). A sensitivity analysis measured the chamber’s response to different source geometries. An authorized physicist independently reviewed these results regularly. AtomLab™ 500 (BIODEX, Mirion Technologies, NJ, USA) re-entrant chambers were used for Lutathera and Pluvicto measurements calibrated with a National Institute of Standards and Technology (NIST) traceable dose provided by the drug manufacturer. The dose measurement accuracy was verified on multiple days against decayed activity.

### 2.2 Hardware and software for dosimetry integration

#### 2.2.1 SPECT/CT system

GE Discovery SPECT/CT 670DR™ (GE Healthcare, Cleveland, USA) is equipped with a SPECT camera, a collimator (low, medium, or high energy, depending on the isotope), a BrightSpeed Elite 16-slice CT scanner, and a Xeleris™ workstation. The Discovery 670 Gamma Camera detectors cover an imaging area of 20 cm in length and 27 cm in width, featuring a 3/8ʺ crystal thickness and a voxel size of 4.42 × 4.42 × 4.42 mm (20). All quality control tests were performed before commissioning as outlined in the AAPM TG 39, 66, and IAEA Report 36 for CT and SPECT/CT systems (21–25). Emphasis was placed on the alignment checks between CT and SPECT scanners to maintain the integrity of image registration for ensuring reliable imaging for dosimetric purposes (26, 27). No generally accepted criteria for alignment accuracy exist, but an alignment mismatch of less than the size of a SPECT image pixel was deemed acceptable (25).

We established a protocol for scanning ^177^Lu-based treatments according to the MIM SurePlan™ MRT Acquisition and Reconstruction Parameter document (a white paper for setting up the MIM SurePlan™ MRT). The main energy window was defined as 208 keV ±10% (187.6 to 229.2 keV); the upper scatter energy window was set to 240 keV ±5% (228 to 252 keV), while the lower scatter energy window was 177.8 keV ±5% (168.9 to 186.7 keV). The image processing and registration module from Xeleris® was utilized for registration since changing bed positions between NM and CT scans does not auto-trigger registrations. Iterative SPECT reconstruction improves accuracy through geometric and intrinsic response corrections. Attenuation artifacts can be corrected using attenuation maps or transmission scans. Hybrid SPECT/CT devices provide high-quality CT scans for attenuation correction and dual or multiple energy windows approaches to correct scatter (28).

A Volumetric Quality Control (VQC) test (which can be replaced by an equivalent vendor-prescribed test) was performed by placing point sources on the scanning system after any significant services on the system to ensure that the table, NM, and CT gantry on the SPECT/CT scanner are aligned correctly (29). Following the raw image acquisition, precise shifts were calculated based on voxel positions. The VQC corrections thus obtained were saved in the data acquisition system. VQC registration corrections were applied before the NM and CT data were exported as a co-registered data set to Xeleris® (19, 30). The Center of Rotation (COR) correction was the final step, which corrects all mechanical and radial misalignment. The COR correction was quantified in the axial direction on every projection as lateral misalignment (25).

#### 2.2.2 Patient-specific dosimetry system integration and commissioning process

MIM SurePlan™ MRT software was evaluated for the feasibility of integrating patient-specific dosimetry into the current RPT program using end-to-end testing. The conversion of the image to dose is accomplished by calibrating the volume sensitivity of the SPECT imaging system in cps/MBq by scanning a cylindrical phantom with a known activity concentration and measuring the counting rate in counts per second (cps). We used a Jaszczak Phantom with 2.5 µM EDTA (ethylenediaminetetraacetic acid) solution prepared with distilled water (31). The EDTA solution was used to prevent activity adhesion to the phantom walls. EDTA solution was mixed with a known amount of Lutathera doses. MIM Sureplan MRT calculated the count rate based on the SPECT images of this phantom. The system volume sensitivity was calculated by dividing the count rate by the activity concentration. Based on the sensitivity factor (cps/MBq), the activity measurement in the area of interest was converted to the appropriate units (25, 28).

NEMA Phantom is widely recognized for SPECT/CT and PET scanner performance evaluation, including verifying dosimetric calculations, characterizing software, and evaluating registration accuracy (32). We utilized it to commission the patient-specific dosimetry system. Lutathera dose mixed with 50 cc of 2.5 µM EDTA solution added to distilled water for uniform dose distribution was injected into the spheres of the phantom. In contrast, the rest of the phantom’s body was filled with the same solution but without radioactivity. The phantom spheres were each filled with 0.13 mCi (22 mm sphere), 0.27 mCi (28 mm sphere), and 0.63 mCi (37 mm sphere) activity. The protocol described in Section 2.2.1 was used to create SPECT images with 4.42-mm isotropic voxels. The GE 16-slice helical scanning system produced CT images with voxel sizes of 0.98 × 0.98 × 3.75 mm. After applying corrections, the co-registered images were transferred from Xeleris® to the MIM® SurePlan™ MRT platform for evaluation. Registered images were checked for alignment between the two modalities. Using software tools, contours for the visualized spheres on each CT scan were outlined. A margin of 10 mm was added to these contours to accommodate the Partial Volume Effect (PVE) observed in SPECT images (33). The software was enabled to convert counts to cps. The counts from each sphere were converted to dose using the calibration factor (CF) calculated by the MIM Sureplan MRT system, yielding activity in mCi. The percentage difference between injected and image-converted activity was calculated for different injected doses in the individual spheres.

## 3 Results

### 3.1 Review and updates on the existing RPT program

**Figure 1:**
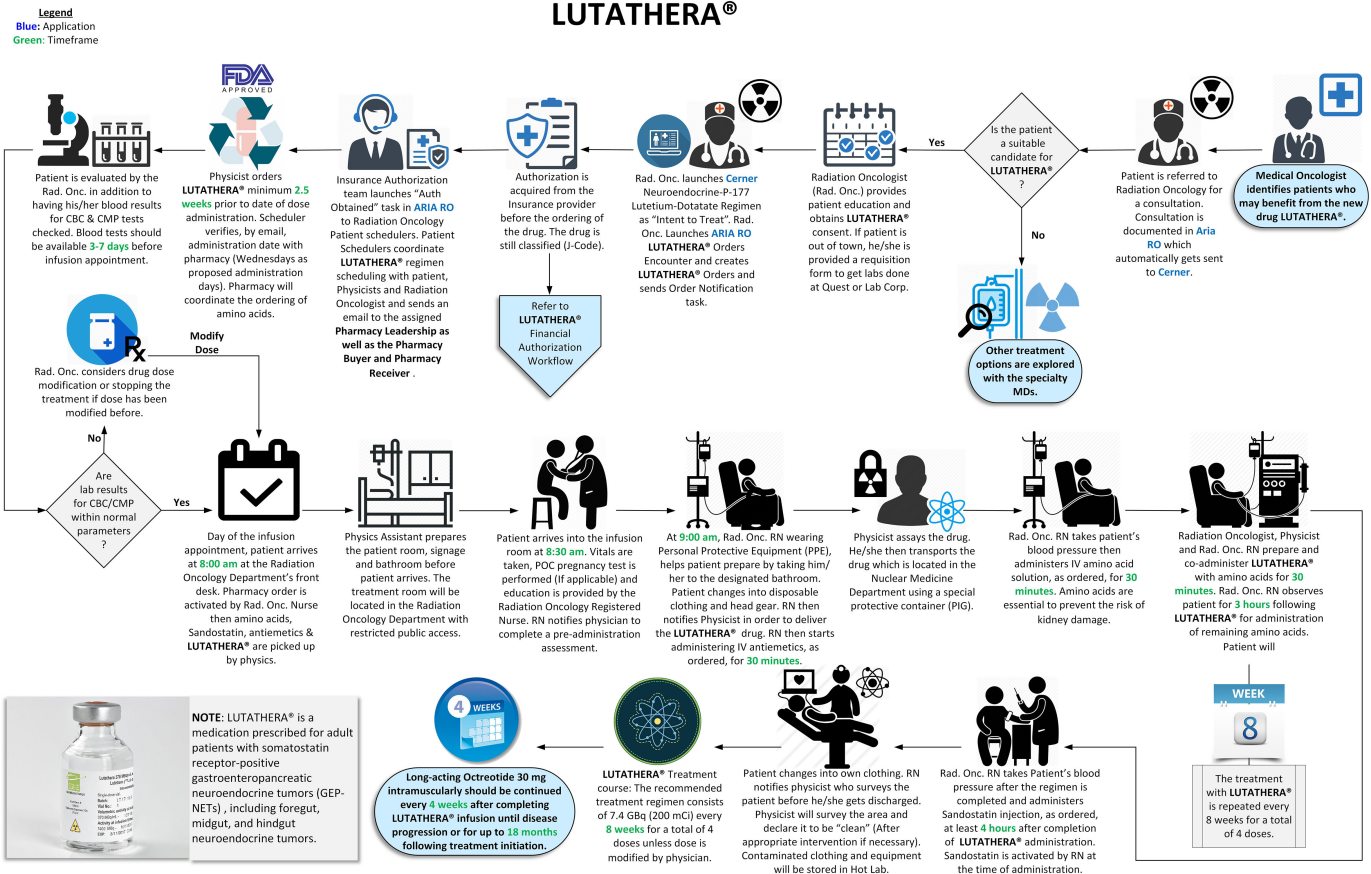
Lutathera administration workflow (34).

**Figure 2:**
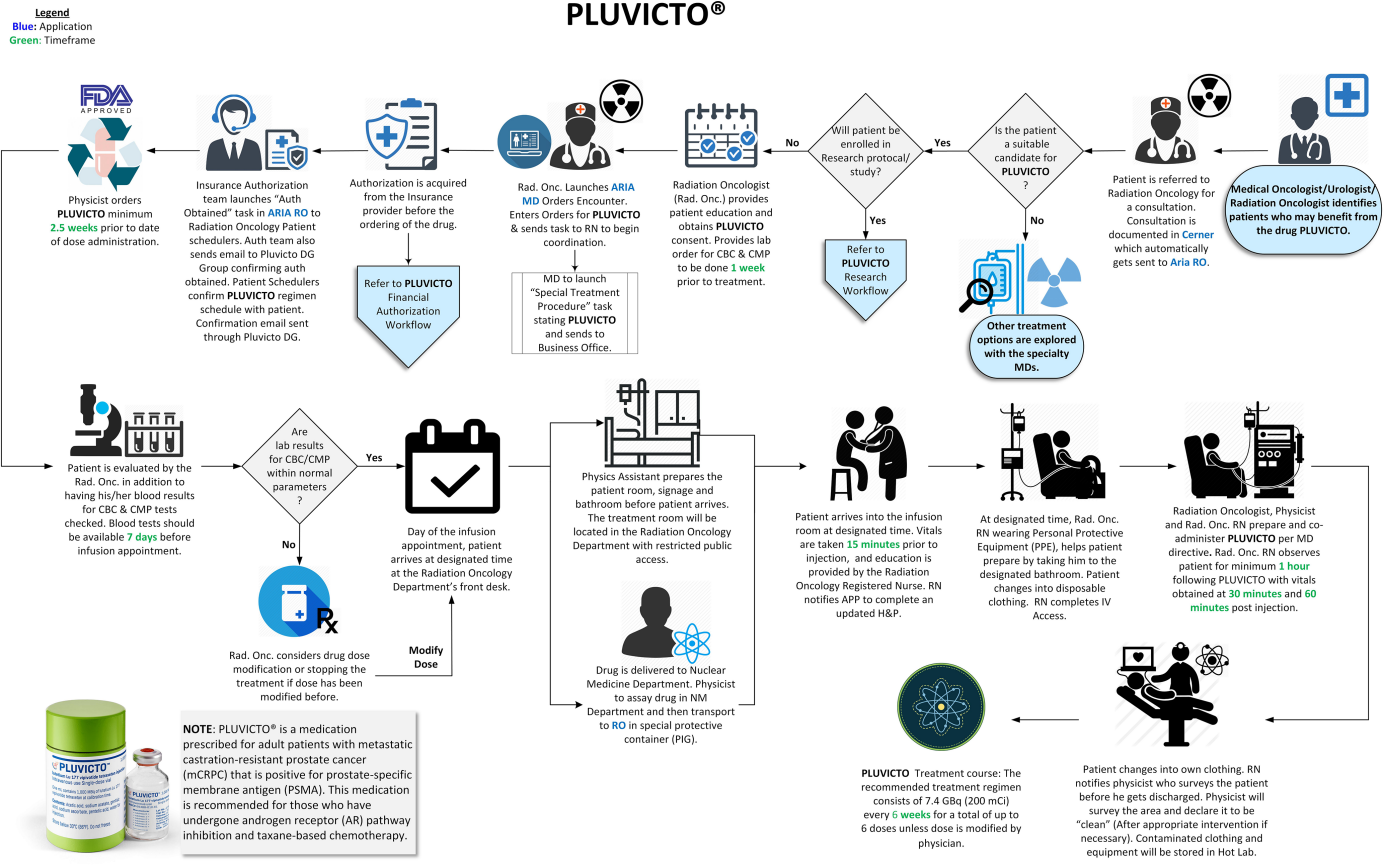
Pluvicto administration workflow.

**Figure 3:**
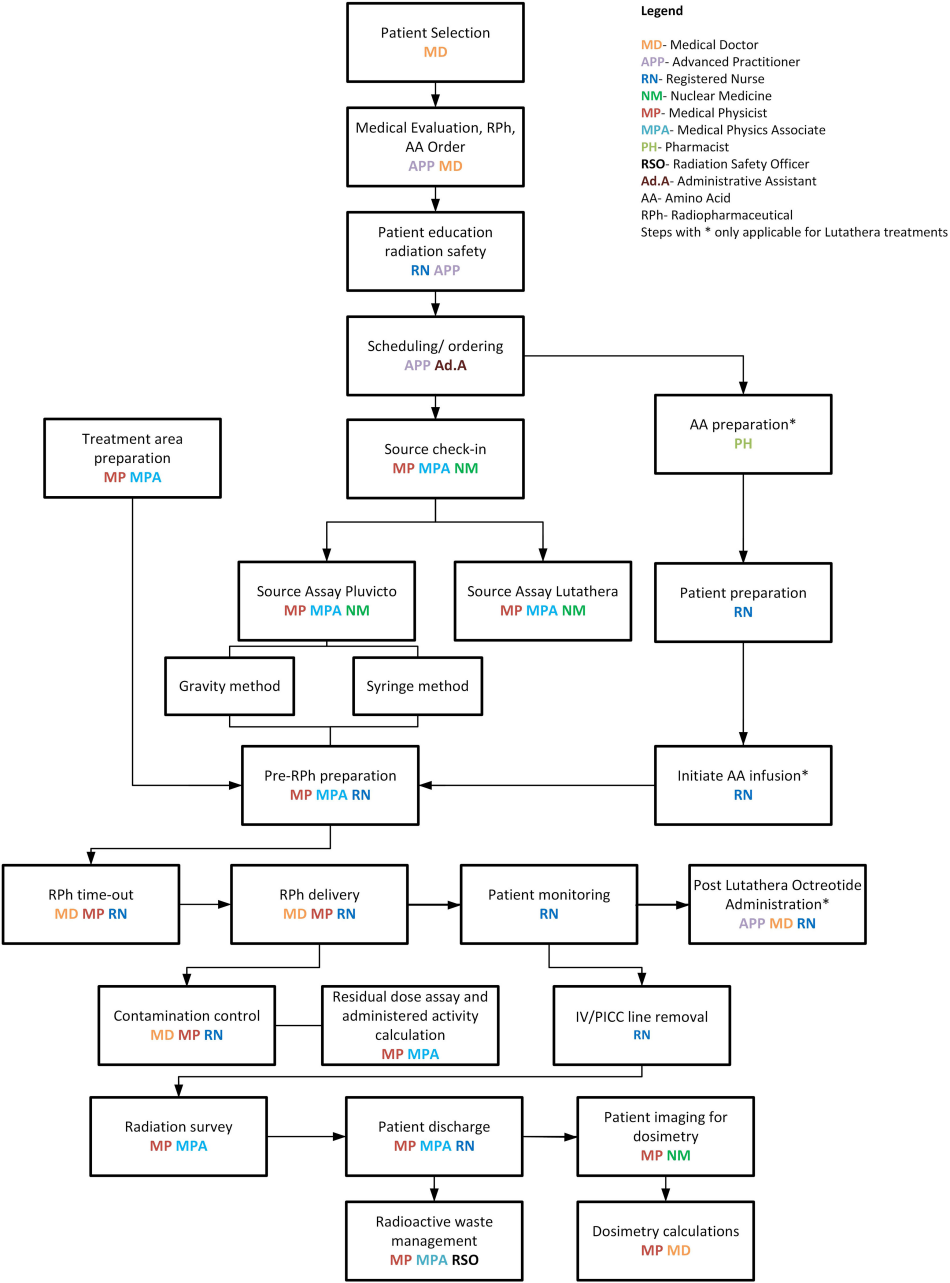
^177^Lu-based treatments and dosimetry—comprehensive workflow.

A seamless program was implemented with continuous quality improvement guiding us while working closely with the imaging department. Robust quality assurance measures were introduced throughout the clinical workflow, from patient selection to post-treatment imaging. Established regulatory guidelines were followed in the designed workflows (**Figures 1**, **2**), with treatment day checklists (**Appendices A and B**) to ensure procedural accuracy. These checklists are indispensable in maintaining consistency and adherence to the highest standards. Moreover, we rely on a rigorously crafted flowchart for “comprehensive Lu-based Radioligand Therapy” as illustrated in **Figure 3**, which serves as a visual guide through the intricate process, ensuring each treatment facet is executed precisely.

#### 3.1.1 Dose calibrator configuration for different geometry

**Figure 4:**
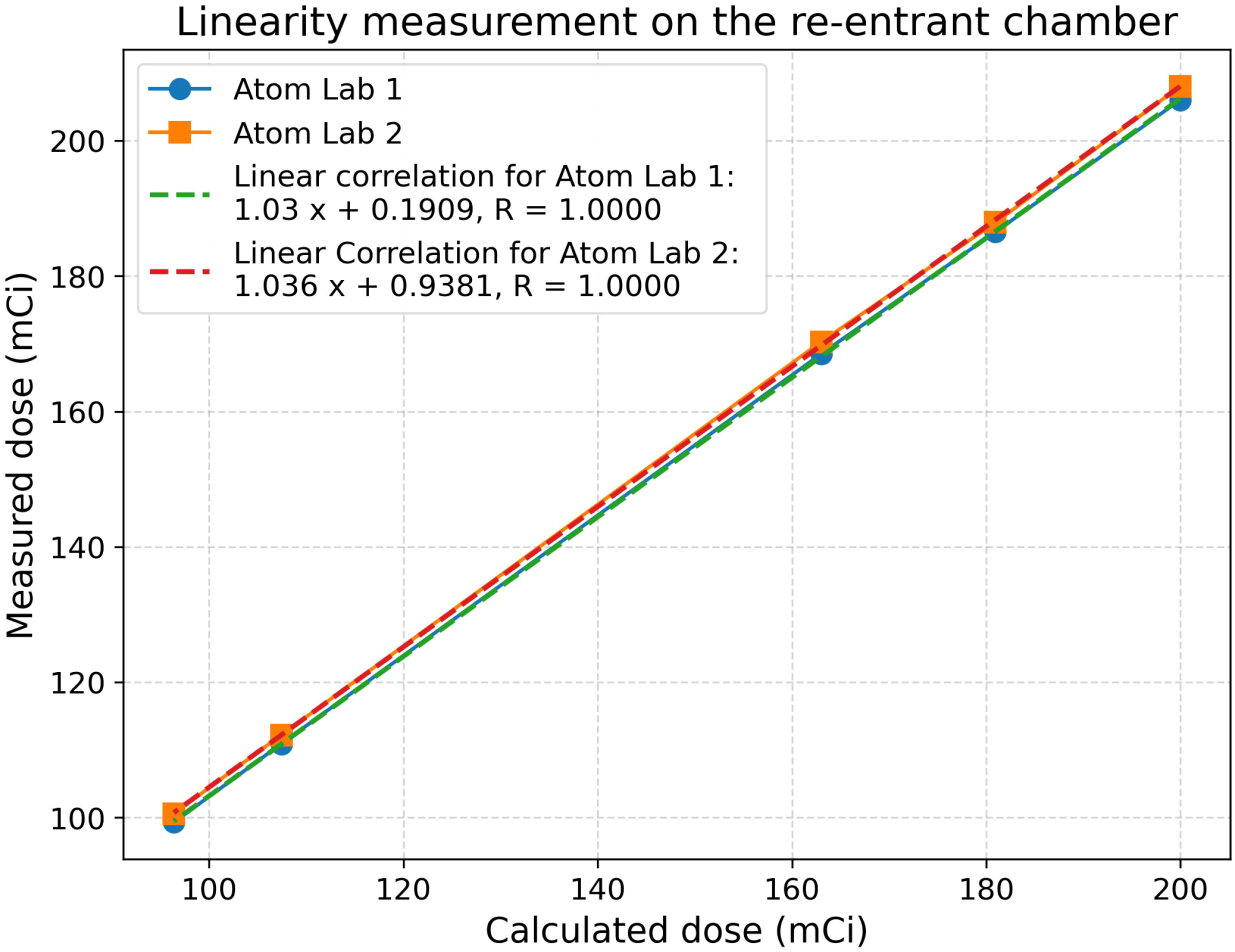
Re-entrant chamber constancy and linearity check; *R*-value indicates the correlation coefficient.

**Figure 5:**
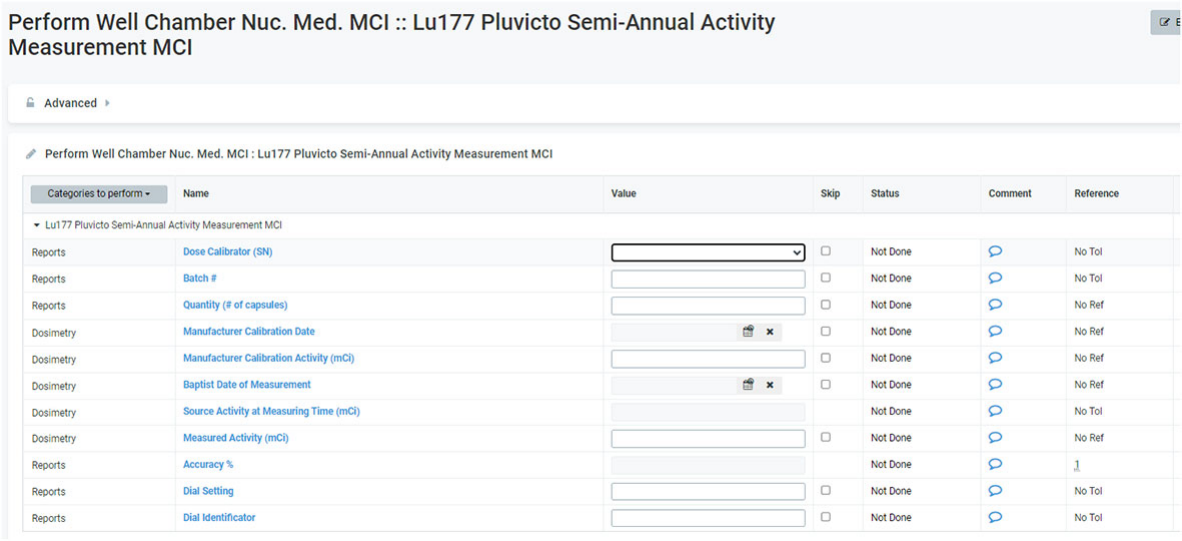
Re-entrant chamber semi-annual QA setup.

The Nuclear Medicine quality assurance checklist indicates that daily consistency checks, quarterly linearity checks, and sensitivity analyses for commonly used radioisotopes are carried out with appropriate approvals. **Table 1** shows constancy and linearity checks performed before Lutathera treatments in the clinic. A linearity check for Lutathera displayed a perfect correlation coefficient of 1.0, as seen in **Figure 4**. Both Lutathera and Pluvicto dose calibration settings were applied to the re-entrant chambers before clinical implementation by using vendor-provided NIST traceable calibration samples. Separate calibration factors are necessary to quantify Pluvicto dose since radioactivity concentration and vial kind are different between the two. Periodic measurements are conducted for specific radiopharmaceuticals with NIST traceable activity to verify the accuracy of dose calibrator performance. “Re-entrant chamber Semi-Annual QA setup” displayed in **Figure 5** uses the same method as the initial re-entrant chamber calibration that guarantees accuracy and consistent performance.

**Table 1:** Lu-177 re-entrant chamber dose calibration and constancy verification: calculated dose vs. re-entrant chamber readings.

Vial no.	Vol (mL)	Calibration time	Activity at calibration time (mCi)	*T*_1/2_ (d)
10	24.500	10/22/18 12:00	245.270	6.6500
		Serial number	Key setting	Key identifier
Atom Lab 1		16111426	114	LU177
Atom Lab 2		16111440	114	LU 177
Date and time		Calculated dose (mCi)	Measured dose (mCi) - % Difference	
			Atom Lab 1	Atom Lab 2
10/24/18 11:00		199.97	206 (3.02)	208 (4.02)
10/25/18 10:00		180.95	186.5 (3.07)	187.9 (3.84)
10/26/18 10:00		163.03	168.5 (3.36)	170.3 (4.46)
10/30/18 10:00		107.43	110.9 (3.23)	112.2 (4.44)
10/31/18 11:20		96.37	99.3 (3.04)	100.6 (4.39)

### 3.2 Hardware updates and software configuration

#### 3.2.1 SPECT/CT system

**Figure 6:**
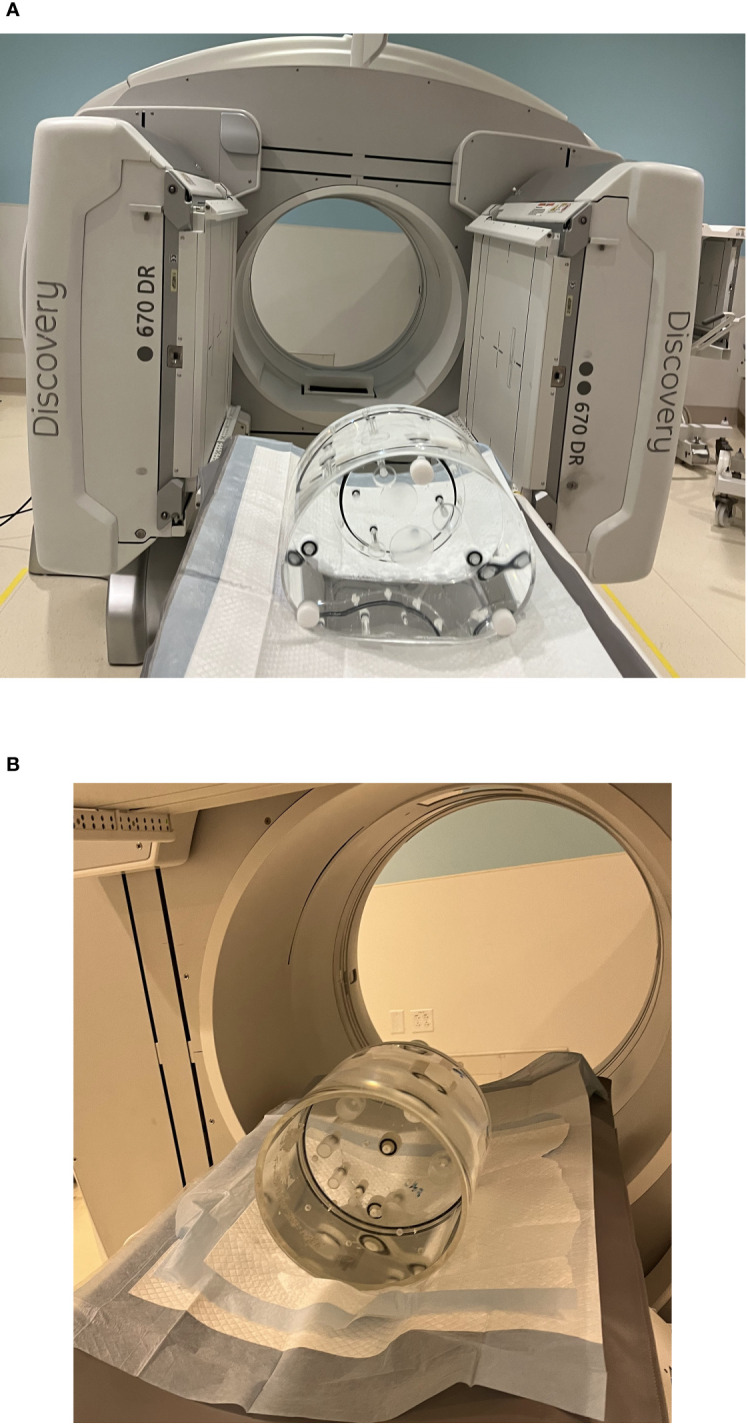
Phantom preparation for RPT and software characterization. **(A)** NEMA phantom scanning setup. **(B)** Jaszczak SPECT phantom scanning setup.

**Figure 7:**
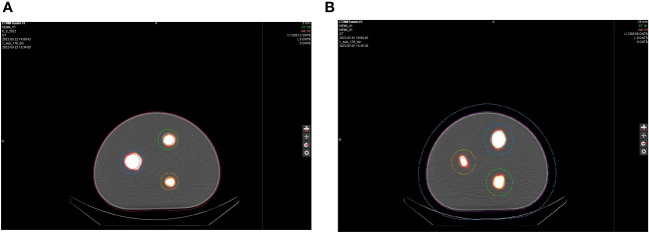
Axial view of the NEMA phantom scans **(A)** NEMA P1 before and **(B)** NEMA A1 after alignment of the hybrid imaging system.

**Figure 8:**
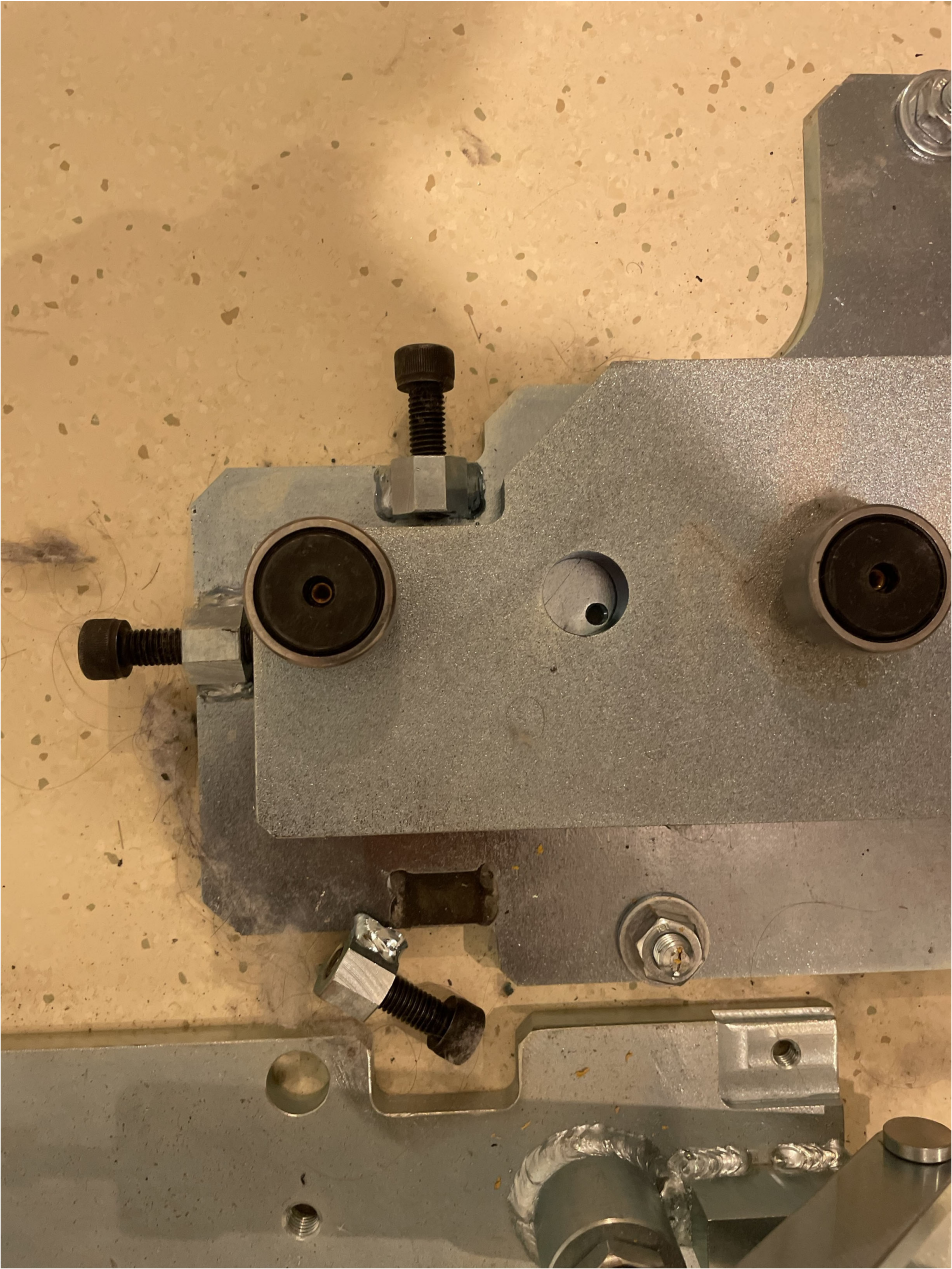
GE SPECT/CT floor-plate replaced for platform stability before aligning the hybrid imaging systems.

Initial phantom images with the medium-energy general-purpose (MEGP) collimator, revealed an image registration discrepancy in the hybrid system's SPECT and CT images. The images obtained using the NEMA Phantoms were evaluated during the software characterization (**Figure 6A**). The radioisotope opacities of CT and SPECT scans differed by more than 5 mm, as displayed in **Figure 7A**. To ensure accurate hardware and software commissioning, we measured SPECT/CT registration deviation and corrected any discrepancies. The service engineers performed the “X-ray to SPECT Registration” accuracy check and the “COR calibration check,” QA tests designed by the GE quality management guidelines to verify image registration accuracy. Each collimator exhibits distinct CT to SPECT registration before software correction factors are applied in the X, Y, and Z directions between the two imaging modalities. The low-energy high-resolution (LEHR) collimator demonstrated acceptable differences (less than 1 mm) in all directions. In contrast, the MEGP collimator displayed significant discrepancies in the X (3.04 mm), Y (0.87 mm), and Z (1.24 mm) directions with the correction factors from LEHR. This exceeded the recommended tolerance of 2.2 mm on any individual axis. Upon review, the MEGP collimator was not subjected to the alignment tests (35). Owing to the magnitude of the discrepancy, there was a need for an extensive service intervention for rectifying the positional alignment of CT with the SPECT system, including replacing a broken floor plate (which affected the platform stability, **Figure 8**) and physically moving the gantry for improved alignment of the two coordinate systems. Upon completion of this, agreement improved, resulting in much smaller discrepancies, X (0.82 mm), Y (0.63 mm), and Z (0.91 mm), as listed in **Table 2**. The COR corrected data from Xeleris® software resulted in registration corrections of 0.05 mm in X, 0.06 mm in Y, and 0.0 mm in Z directions for the MEGP collimator verified using independent software. The improvement of the alignment of CT to SPECT images can be seen in **Figure 7B**.

**Table 2:** SPECT/CT registration QA results before and after alignment of the hybrid-imaging system evaluated with “COR calibration check” and MEGP collimator.

Date	X	Y	Z
	Mean (Standard Deviation)		
5/2/2022	**3.04 (0.95)**	0.87 (0.48)	1.24 (0.68)
6/6/2022	0.82 (0.39)	0.63 (0.45)	0.91 (0.06)
Limit Specs	2.2		

CT to NM difference in mm. Out-of-tolerance values are in bold letters.

#### 3.2.2 Integration of the patient specific dosimetry system

The vendor and hospital IT department collaborated to integrate MIM SurePlan™ MRT into the clinical environment. Computational resources and software installation were carefully evaluated for successful implementation. MIM Sureplan MRT software can be installed on a local workstation or on the MIMcloud®, or integrated with other commonly used MIM suites, such as MIM Maestro, according to the technical requirements of the department (36). We installed the software on a local workstation. Images obtained from Jaszczak Phantom measurements discussed in Section 2.2.2 (**Figure 6B**) were exported to MIM SurePlan™ MRT software. A CF of 5.04159 cps/MBq was obtained based on the initial image set before the field service intervention and 5.124969 cps/MBq based on the image set collected after the field service.

To evaluate the dose in the spheres of the NEMA Phantom image set, MIM Sureplan MRT was used. The 37-mm sphere yielded 251.6 counts, converting to 1.33 mCi, resulting in an 11.8% difference between the injected and the estimated activity. The medium sphere showed a 12.3% difference (109.5 counts resulting in 0.58 mCi dose), while the small sphere showed an 8.9% difference (51.5 counts converting to 0.27 mCi dose).

## 4 Discussion

This study describes the practical considerations and basic tasks necessary to perform SPECT-based dosimetry in an RPT clinic that treats high volumes of Lutathera and Pluvicto patients. Department-specific checklists and workflows are essential for ensuring safe and effective RPT. We analyzed and updated our department’s workflow using the PDCA tool, enhancing the efficiency and safety of the ^177^Lu-based RPT. The re-entrant chamber calibration should be performed using a NIST-certified activity using a vial that will be used for patient dose assay, with the vial placed at the exact location in the re-entrant chamber where routine dose assay will be performed. Thus, each radiopharmaceutical will store a unique calibration factors in the device for future use. The hybrid SPECT/CT scanner enables simultaneous visualization of the two image datasets linked in precise spatial–temporal coordinates. This facilitates improved diagnostic interpretation since CT scans provide anatomical context and SPECT provides uptake information, besides applying CT attenuation coefficient corrections to the SPECT data, which is necessary for dosimetric calculations (26, 27). The SPECT/CT registration test, also known as the COR calibration check, is designed to evaluate the alignment of SPECT images with CT images obtained with the appropriate collimator used for the specific radiopharmaceutical. A VQC test or equivalent is used to evaluate the agreement of the two datasets after applying digital corrections. If these corrections need revision for improved agreement, they should be addressed at this time and stored for future use. Typically, these are minor geometrical corrections applied to the SPECT data. MIM Sureplan™ MRT software was used for dose conversion and end-to-end tests, employing commonly available Jaszczak and NEMA Phantoms.

**Figure 9:**
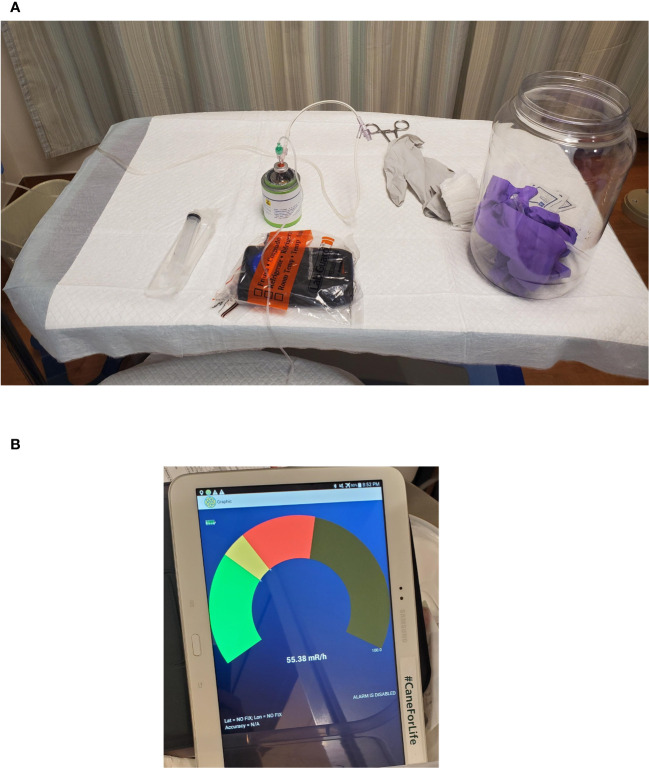
Survey Meter Setup for remote display of the instantaneous dose rate of the dose flow during RPT infusion. **(A)** Treatment cart with survey meter arrangement for remote display. **(B)** Remote Bluetooth display.

Although many of the tests described above form the bulk of commissioning, a robust program ensuring a smooth dose delivery is essential. Ensuring patient safety and minimizing radiation exposure is paramount while administering ^177^Lu-based treatment. In addition, it is essential to reduce radiation exposure to medical personnel and anyone else who may come into contact with the patient during or after the treatment. We accomplished this goal by following institutional policies and existing radiation protection regulatory requirements, radioactive material license agreements, Nuclear Regulatory Commission (NRC) guidelines for patient release (37, 38), and instructions provided to the patient for follow-up radiation protection (15, 39, 40). During Lutathera and Pluvicto treatments, we monitor the radioactivity levels from a distance to reduce exposure to the staff, using a digital radiation monitor and Bluetooth display (**Figure 9**). Continuous monitoring for dose spillage, extravasation, or a block in the dose transportation catheters is vital during RPT infusion and should be emphasized. Following the administration of the RPT, the patients should empty their bladder and undergo radiation exposure surveys at the waist level before leaving the department. Surveys can be used to monitor radioactivity retained in the kidneys and bladder. With the experience of delivering Lutathera using an infusion pump, we devised a similar workflow, pumping the Pluvicto dose to the patient in 15 min, illustrated in **Figure 2**. Zoberi et al. present a comprehensive account of using a syringe method to dispense Pluvicto (41). The staff should receive proper training to prevent contamination or spillage when administering Pluvicto via syringe. Incontinent patients receiving Pluvicto treatment for advanced prostate cancer may use a Foley catheter to avoid the possibility of inadvertent leakage of urine resulting in radiation contamination.

RPT dosimetry is mandatory in Europe but is limited to patient-averaged dosimetry, and it has yet to be implemented in other countries. Whether there are enough clinical data to guide RPT treatment planning or ample clinical benefit of dosimetry for ^177^Lu-based RPT is an ongoing debate. There is concrete clinical evidence for ^90^Y radioembolization, another widely used RPT, that dosimetry is beneficial for personalized treatment planning (42). It is necessary for emerging RPT programs to incorporate successful personalized dosimetry methods to improve benefits by individualized treatments deviating from the conventional empirical method of delivering a fixed dose to all patients (10). Personalized dosimetry for ^177^Lu-based RPT is a challenging task that is yet to be clinically proven. Standard RPT regimens have already been validated in clinical trials with a favorable risk–benefit balance, and the pursuit of clinical benefits from dosimetry may need more evidence of improved efficacy (43). According to Götz et al., the total relative error in dose estimation can amount to 25.0%, which includes an error in time-integrated activity (17.1%), S-value error (16.7%), segmentation error (5.4%), and activity measurement-related inaccuracy (5.0%) (44). The FDA approval of Pluvicto and Lutathera was based on the Phase III trials that used a “safe dose” of 200 mCi per fraction to ensure that the cumulative kidney tolerance is not exceeded by the complete course of treatment, which could result in less-than-optimal outcomes for some patients. However, this safe dose estimate, tested and proved by clinical trials to be optimal for treatments, allowed many patients to benefit from these newly approved RPTs based on ^177^Lu. Personalized dosimetry is not a requirement for administering FDA-approved Lu-based treatments. Still, it is considered helpful since these treatments can lead to cessation and prescription changes due to side effects, including myelosuppression and renal toxicity. ^177^Lu exposure can cause organ damage as kidneys eliminate it (45, 46). PSMA-targeted therapy risks include xerostomia and nephrotoxicity (2). Multiple studies evaluated the absorbed doses and the correlation between dose and response in tumors treated with ^177^Lu-RPT (8, 10, 47, 48). Higher absorbed doses lead to better tumor response, but variations in binding affinity, receptor density, hypoxia, and tumor volume can affect the outcome. While dose–effect relationships have been demonstrated, dosimetry is not the sole factor influencing treatment outcomes (49).

Commercial software programs are available for patient-specific dosimetry of ^177^Lu radiopharmaceuticals, which use a range of scientific principles to achieve respective objectives. These programs employ different dosimetry methods, utilizing data from hybrid imaging systems (11, 12, 14). Using radioactivity deposition images, patient-specific dosimetry software locates and analyzes dose delivery details, toxicity to OAR, and the absorbed dose to the target (50, 51). MIM SurePlan™ MRT software uses voxel-based calculations to compute the dose delivered by a radionuclide to the target voxel, generating a dose map and statistical dose value curves. The VSV approach calculates the dose in water, where sources and targets are represented by voxels distributed in the volume of interest. Before any commercially available software is used for dosimetry quantification, a specialist must thoroughly evaluate, examine, and test it. Sufficient planning and time allocation are necessary to implement radiopharmaceutical dosimetry software successfully in a clinical environment. Accurate dosimetry requires the imaging system and dose calculation software to be integrated and tested thoroughly using phantom studies. Workflow optimization should be completed through iterated revisions and feedback from user interactions. RPT patient-specific dosimetry should be conducted under authorized users’ supervision, with an appropriate understanding of the imaging and dosimetry system.

Spatially aligned SPECT/CT images provide accurate dose estimates based on the activity concentration within tumors and OAR. This is particularly beneficial in oncology, as specific radiopharmaceuticals target tumor physiology. The use of quantitative SPECT in oncology is expanding with radionuclide therapies (25, 27). In many institutions that primarily use hybrid imaging systems for diagnostic purposes, calculating the absorbed dose is not usually considered a requirement during the acceptance process. Additionally, an appropriate quality management program to support dosimetry calculations may not be considered essential. However, establishing a solid dosimetry program necessitates a comprehensive evaluation and validation process for the imaging system, image reconstruction algorithm, and dosimetric calculation software. In hybrid imaging systems, dose calculation and reporting inaccuracies can arise due to misaligned NM and CT images. SPECT/CT misalignment can lead to errors in patient-specific dosimetry, affecting dose coefficient calculation for tumors, OAR, and the whole body.

Accurate alignment is essential to avoid mistakes in attenuation-scatter correction, partial-volume averaging, tissue overlapping, and biodistribution and clearance measurement (11). Dose calculation accuracy can be improved, and the overall dosimetric uncertainties can be reduced by improving imaging techniques, such as spatial alignment accuracy, improving VOI delineation, and removing uncertainties in dose assay (44). A significant reduction in SPECT/CT imaging disagreement is feasible with available techniques and vendor-supported machine service. Hybrid imaging disagreement was reduced from approximately 5 mm to <1 mm as a result of this study. The dose conversion calculation obtained by multiplication of cps/mL*cc and the CF verified the dose accuracy within ±12% in the spheres of the NEMA Phantom. These can be further improved by utilizing improved dose kernels in the software and conducting multiple time point (MTP) scans. This contrasts the single SPECT/CT imaging we conducted in this specific study, which aimed solely to assess the feasibility of integrating a patient-specific dosimetry program for ^177^Lu-based RPT into clinical practice.

Performing MTP scans for patient-specific dosimetry can be impractical and demanding on patients and healthcare departments. Despite MTP’s superior technical merits, the main obstacle to dosimetry in the US is the difficulty in returning patients for imaging after the procedure due to reimbursement issues and patient convenience concerns. Recent developments in dosimetry with single time point (STP) imaging studies provide reasonable accuracy in dosimetry output and convenience for patients and clinicians (52, 53). Ha¨nscheid et al. reported approximately 10% deviation in dose accumulation values at the tumor and different OARs on dosimetry uptake calculations with MTP image sets from six different time points (24, 48, 72, 96, 120, and 144 h) compared against STP data at 96 h (54). In a study to improve patient comfort and reduce the costs of ^177^Lu-DOTATATE treatments, one SPECT/CT image was taken one day or seven days after treatment to perform dosimetry. The results showed that the one day images were accurate for kidney doses, while the seven days images were more accurate for tumors (55). Another study showed that performing one SPECT/CT scan one day post-injection increases uncertainty by approximately 19% (56). Optimizing the dosimetry protocol for ^177^Lu-PSMA therapy highlighted the importance of accurate lesion dosimetry. SPECT imaging at 168 h yielded approximately 20% accurate results, but including a second-time point reduced the uncertainty to a comparable level to the reference standard of 14% (57). These studies show that STP and MTP have a reasonable correlation for all tissues but that there are outliers for which dosimetry assessment is different if dosimetry calculations are performed using STP only. Even with MTP, integration based on a limited number of time points could lead to errors. Although STP does not generate bias, estimations based on patient-specific data differ by several tens of percent between STP and MTP. STP has yet to be an established method for patient-specific dosimetry.

Patient-specific dosimetry calculations for RPT in clinical settings are complex and require careful consideration of technical requirements, evaluation of dose calculation methods, and understanding of regulatory and reimbursement issues. Factors such as time, resource intensity, training, and education play vital roles. Evaluating the financial feasibility of utilizing the software to enhance patient care is essential for a successful implementation (58). Different studies quantitatively evaluate the SPECT/CT imaging camera for performance using different reconstruction algorithms. Some assess recovery coefficients and spatial resolution in a NEMA IEC phantom with different sphere-to-background ratios for determining ^177^Lu activity concentration (59, 60). Another study proposes incorporating patient dosimetry into clinical practice with recovery coefficient determination with various phantoms (61). Only three spheres of the NEMA phantoms were filled in our study, as injecting the radiopharmaceuticals was challenging for the smaller spheres. Different activities were present in three spheres as we injected uniformly distributed dose solutions into the different volume spheres. Information collected from the three spheres was sufficient to review the spatial geometry accuracy of the hybrid imaging system.

SPECT/CT image quality assessment is only one minor aspect of the dosimetry program setup, which may be a fundamental step in integrating dosimetry with existing RPT programs. Our study discusses the errors introduced by the imaging system alignment and recommends practical resolutions for an end user to start the RPT dosimetry program; we did not address the details of characterizing or analyzing the RPT dosimetry with recovery coefficient or any other dosimetry accuracy evaluation techniques in this study, which is difficult to accommodate in this paper and will follow in future studies with in-depth analysis of the collected images. Characterization, algorithm validation, comparison, or independent verification of dose calculations with MC simulations for different tissues are necessary to evaluate clinical MRT software comprehensively. A detailed analysis of each step of the treatment workflow is consequential in identifying and preventing potential failures. Failure mode and effects analysis (FMEA) is a fundamental methodology for identifying possible failure modes and implementing preventive measures. Despite its extensive use in various specialties for risk assessment, only a few studies have applied FMEA to RPT (39, 62, 63). A multi-institutional research incorporating fault tree analysis will identify potential failure modes so that the severity and likelihood can be evaluated and measures to mitigate risks can be undertaken, as described in AAPM’s TG-100 recommendations (64).

## 5 Conclusion

This paper presents workflows and checklists for setting up Pluvicto and Lutathera RPT programs to ensure program quality, smooth delivery, and completeness. The existing ^177^Lu-based RPT program is reviewed in detail, and our clinical observation for practice improvement is shared. Our experience with clinical feasibility evaluation and commissioning of an RPT dosimetry software is discussed. We resolved image registration issues on the SPECT/CT system. We provided a detailed discussion of the hybrid imaging registration process, essential for integrating patient-specific dosimetry into an existing RPT program.

^177^Lu-DOTATATE individualized treatment based on renal dosimetry is feasible with low toxicity, showing promising efficacy that can improve results beyond a standard approach, and should be further evaluated in randomized trials (48). There is a significant potential to safely increase the radiation dose to lesions during the early stages of peptide receptor radionuclide therapy (PRRT) cycles due to the higher radiation dose delivered during the first treatment in PRRT cycles (49). However, only a properly designed clinical trial can provide a definitive answer. Ongoing ^177^Lu RPT trials highlight the importance of customized regimens via patient-specific dosimetry (14). Establishing a standard procedure for dosimetry calculations in nuclear medicine is essential for advancing personalized medicine. With the rapid development of information technologies in medicine, dosimetry programs will likely become integral to the standard gamma camera software (65). Personalized dosimetry could encourage oncologists to consider alternative treatment options. Providing versatile tools and appropriate methodology for dosimetry implementation in clinical practice can pave the way for broader adoption and improved personalized dosimetry methods in this rapidly growing but somewhat nascent therapeutic modality. To implement a reliable dosimetry program, assessing the current RPT program thoroughly, verifying the imaging system’s integrity, ensuring accurate image reconstruction, and seamlessly integrating the software used for dosimetric calculations with appropriate quality assurance at every stage are essential.

## Data availability statement

The original contributions presented in the study are included in the article/supplementary material. Further inquiries can be directed to the corresponding author.

## Author contributions

SG: Conceptualization, Data curation, Formal analysis, Resources, Writing – original draft, Investigation, Project administration, Software, Validation. RT: Methodology, Writing – review & editing. SA: Data curation, Software, Visualization, Writing – original draft. TK: Methodology, Validation, Writing – review & editing. ES: Supervision, Writing – review & editing. VM: Methodology, Writing – review & editing.

## Appendix

### A: Lutathera Treatment Day Checklist

- Place appropriate radiation signs (restricting public access).
- Procedure room and restroom preparation with floor covering to contain any potential contamination.
- The patient arrives at the procedure room with a suitable escort.
- The patient is positioned on an infusion chair or stretcher according to the arrangement.
- Initiation of the patient encounter in Electronic Medical Record (EMR) system.
- Review and update radiation protection instructions with the patient, conduct the patient assessment, discuss expectations, and prepare medications.
- Discuss toilet usage instructions with the patient (sitting on the seat while using the toilet and placing the chuck lining above the commode when flushing the toilet to avoid radiation contamination).
- Appropriate IV (Intravenous) line placement or PICC (Peripherally Inserted Central Catheter) infusion line preparation.
- Completes the anti-emetic injection.
- Starts the amino-acid (AA) injection (250 mL/hour).
- Check the flow on the lines to see any resistance for possible bolus on the skin.
- Prepare the needles that go to the Lutathera dose vial and check the flow using the tri-connector (the tri-connector is removed after the flow is verified).
- Program the infusion pump’s flow rate for saline (50 mL/hour to 100 mL/hour for 5-10 min: 200-300 mL/hour for 20-30 min).
- Patient is asked to use the bathroom to void before Lutathera infusion starts (After 30 minutes of AA injection).
- Assess the room preparation for RPT and Survey meter setup to monitor radioactivity.
- Pre-treatment assay of the dose and bring the dose to the treatment area using transport lead shield.
- Anyone handling radioactive material wears the appropriate personnel protection equipment.
- Insert a tape roll inside the lead shield to lift the vial, place the Lu-177 vial on top of the tape roll, and mark the fluid level outside the vial.
- Flush the infusion line and lock the clamp to ensure a smooth start for Lutathera delivery.
- All involved staff participate in the patient time-out- record in the EMR.
- Verification of long/short needles are connected to the appropriate tubing.
- A long needle should connect the vial to the patient.
- A short needle should connect the Saline bag to the vial.
- Wipe the cap of the Lu-177 vial with an alcohol swab using tongs for 15 seconds.
- Insert the small needle from the saline above the fluid level and the long needle connected to the patient to the Lu-177 vial under the liquid level to the bottom of the vial (bevel up on both needles).
- Gently massage the areas where the clamps were previously locked onto the tubing before starting the pump.
- Start the infusion pump for saline flow and ensure the flow is appropriate and the fluid level is not rising in the vial above the mark.
- Survey any staff before they leave the treatment area and contain any radioactive waste.
- Monitor infusion line for bulging, leakage, or exacerbation to ensure proper pump functionality and prevent dose spillage.
- If the fluid level goes above the mark, stop the pump immediately and do the appropriate intervention. A stop to Lutathera should also include a stop to amino acids.
- Gently push the remaining fluid in the vial by injecting air (air phase) to complete the Lutathera infusion in 30 min.
- Disconnect the saline line from the short needle base and the Lu-177 line from the patient sequentially. Connect the Lu-177 line to the short needle’s base to create a closed loop with both needles fixed on the vial. Place the closed-loop radioactive waste containing the Lutathera vial, needles, and contaminated line into a hand glove and place it in the transport lead shield.
- Trash any remaining Lu-177 line into the Nalgene container with other contaminated items.
- Take the Lu-177 bottle and the Nalgene container for post-assessment/storage.
- Amino acid infusion continues for three more hours after the Lu-177 injection.
- Patient urinates after RPT infusion and waist-level radiation survey at 1m.
- Post Lutathera Octerotide Injection to the patient is administered.
- Monitor and assess the patient throughout the procedure and prepare for discharge.
- Discuss the future schedule or imaging appointments with the patient or caretakers before discharge.
- Survey the patient at the waist level before release from the facility (Release criteria < 5 mR/hr at 1m).
- Provide the patient with a ‘dose info wallet card’ with information about the dose delivered for use in emergencies and discharge the patient.
- Inspect the treatment area for contamination and restrict access to the room, toilet, and adjacent areas based on radiation levels.
- Complete the EMR encounters and documentation for the treatment.

### B: Pluvicto Treatment Day Checklist- Infusion Pump Method

- Place appropriate radiation signs (restricting public access).
- Procedure room and restroom preparation with floor covering to contain any potential contamination.
- The patient arrives at the procedure room with a suitable escort.
- The patient is positioned on an infusion chair or stretcher according to the arrangement.
- Initiation of the patient encounter in Electronic Medical Record (EMR) system.
- Review and update radiation protection instructions with the patient, conduct the patient assessment and discuss expectations.
- Discuss toilet usage instructions with the patient (sitting on the seat while using the toilet and placing the chuck lining above the commode when flushing the toilet to avoid radiation contamination).
- Appropriate IV (Intravenous) line placement and infusion line preparation.
- Check the flow on the lines to see any resistance for possible bolus on the skin.
- Prepare the needles that go to the Pluvicto dose vial and check the flow using the tri-connector (the tri-connector is removed after the flow is verified).
- Program the infusion pump’s flow rate for saline (250 mL/hour for 15 min).
- Patient is asked to use the bathroom to void before Pluvicto infusion starts.
- Assess the room preparation for RPT and Survey meter setup to monitor radioactivity.
- Pre-treatment assay of the dose and bring the dose to the treatment area using transport lead shield.
- Anyone handling radioactive material wears the appropriate personnel protection equipment.
- Insert a tape roll inside the lead shield to lift the vial, place the Lu-177 vial on top of the tape roll, and mark the fluid level outside the vial.
- Flush the infusion line and lock the clamp to ensure a smooth start for Pluvicto delivery.
- All involved staff participate in the patient time-out- record in the EMR.
- Verification of long/short needles are connected to the appropriate tubing.
- A long needle should connect the vial to the patient.
- A short needle should connect the Saline bag to the vial.
- Wipe the cap of the Lu-177 vial with an alcohol swab using tongs for 15 seconds.
- Insert the small needle from the saline above the fluid level and the long needle connected to the patient to the Lu-177 vial under the liquid level to the bottom of the vial (bevel up on both needles).
- Gently massage the areas where the clamps were previously locked onto the tubing before starting the pump.
- Start the infusion pump for saline flow and ensure the flow is appropriate and the fluid level is not rising in the vial above the mark.
- Survey any staff before they leave the treatment area and contain any radioactive waste.
- Monitor infusion line for bulging, leakage, or exacerbation to ensure proper pump functionality and prevent dose spillage.
- If the fluid level goes above the mark, stop the pump immediately and do the appropriate intervention.
- Gently push the remaining fluid in the vial by injecting air (air phase) to complete the Pluvicto infusion (No time restrictions exist for Pluvicto Infusion).
- Disconnect the saline line from the short needle base and the Lu-177 line from the patient sequentially. Connect the Lu-177 line to the short needle’s base to create a closed loop with both needles fixed on the vial. Place the closed-loop radioactive waste containing the Pluvicto vial, needles, and contaminated line into a hand glove and place it in the transport lead shield.
- Trash any remaining Lu-177 line into the Nalgene container with other contaminated items.
- Take the Lu-177 bottle and the Nalgene container for post-assessment/storage.
- Patient urinates after RPT infusion and waist-level radiation survey at 1m.
- Monitor and assess the patient throughout the procedure and prepare for discharge.
- Discuss the future schedule or imaging appointments with the patient or caretakers before discharge.
- Survey the patient at the waist level before release from the facility (Release criteria < 5 mR/hr at 1m.).
- Provide the patient with a ‘dose info wallet card’ with information about the dose delivered for use in emergencies and discharge the patient.
- Inspect the treatment area for contamination and restrict access to the room, toilet, and adjacent areas based on radiation levels.
- Complete the EMR encounters and documentation for the treatment.
