# Corrigendum: A single-institution experience with 177Lu RPT workflow improvements and qualifying the SPECT/CT imaging for dosimetry

**DOI:** 10.3389/fonc.2024.1410818

**Published:** 2024-04-12

**Authors:** Siju C. George, Ranjini Tolakanahalli, Santiago Aguirre, Taehyung Peter Kim, E. James Jebaseelan Samuel, Vivek Mishra

**Affiliations:** ^1^ Department of Radiation Oncology, Miami Cancer Institute, Baptist Health, Miami, FL, United States; ^2^ Department of Physics, School of Advanced Sciences, Vellore Institute of Technology, Vellore, India

**Keywords:** ^177^Lu treatments, patient-specific dosimetry, clinical implementation, dose calibration, registration error, treatment day checklist, clinical workflow, hybrid imaging system QA

In the published article, there was a mistake in the corresponding author. The corresponding author has been updated to E. James Jebaseelan Samuel, with the email ID `ejames@vit.ac.in.

The authors apologize for this error and state that this does not change the scientific conclusions of the article in any way. The original article has been updated.

